# The potential of using semitendinosus tendon as autograft in rabbit meniscus reconstruction

**DOI:** 10.1038/s41598-017-07166-z

**Published:** 2017-08-01

**Authors:** Chenxi Li, Xiaoqing Hu, Qingyang Meng, Xin Zhang, Jingxian Zhu, Linghui Dai, Jin Cheng, Mingjin Zhong, Weili Shi, Bo Ren, Jiying Zhang, Xin Fu, Xiaoning Duan, Yingfang Ao

**Affiliations:** Institute of Sports Medicine, Beijing Key Laboratory of Sports Injuries, Peking University Third Hospital, 49 North Garden Road, Haidian District Beijing, 100191 People’s Republic of China

## Abstract

Since transplantation of meniscal allograft or artificial menisci is limited by graft sources and a series of adverse events, substitution for meniscus reconstruction still needs to be explored. Natural biomaterials, which can provide a unique 3-D microenvironment, remain a promising alternative for tissue engineering. Among them, autograft is a preferred option for its safety and excellent biocompatibility. In this study, we utilized semitendinosus tendon autograft in meniscus reconstruction to investigate its fibrochondrogenic metaplasticity potential and chondroprotective effect. Tendon-derived stem cells (TDSCs) and synovial-derived mesenchymal stem cells (SMSCs), two most important stem cell sources in our strategy, exhibited excellent viability, distribution, proliferation and fibrochondrogenic differentiation ability in decellularized semitendinosus tendon (DST) scaffolds *in vitro*. Histologic evaluation of the tendon grafts *in vivo* suggested endogenous stem cells differentiated into fibrochondrocytes, synthesized proteoglycan, type II collagen and radial type I collagen at 12 weeks and 24 weeks post-surgery. As for elastic modulus and hardness of the grafts, there were no significant differences between native meniscus and regenerated meniscus at 24 weeks. The protection of condylar cartilage from degeneration was significantly better in the reconstruction group comparing to control group. Overall, semitendinosus tendon autograft seems to be a promising substitution in meniscus reconstruction.

## Introduction

Meniscus tear is one of the most common damages in sports medicine. Given the relative avascular nature and limited self-repairing capability of meniscus, traditional partial or total meniscectomy is one of the most frequently performed orthopaedic surgeries to improve short-term function of the knees for patients with symptomatic meniscus tear^1^. However, progressive osteoarthritis is inevitable as the joints are exposed to dynamic instability in the long term after meniscectomy^2^. Transplantation of meniscal allograft or artificial menisci appears to restore the anatomy construction, relieve symptoms and delay the progression of osteoarthritis^3, 4^. However, allograft meniscus transplantation is limited by graft sources, high clinical failure rate and ethical issues^5–8^, and artificial menisci was reported with adverse events including graft ruptures and shrinkage, limited cellular infiltration and poor mechanical properties^9–13^. Therefore, substitution for meniscus reconstruction still needs to be further explored.

Meniscus, mainly composed of fibrochondrocytes, type I collagen fibers, proteoglycan, a little type II collagen fibers and multiple bioactive factors, is a semilunar fibrocartilaginous tissue with complicated structure^14^. Natural biomaterials with highly complexed micro-structure can provide a unique 3-D microenvironment for stem cells differentiation, and they have drawn increasing attention as a promising alternative in tissue engineering^15^. Tendon tissue has the same orientation of the type I collagen fiber bundles as the peripheral half of the meniscus, suggesting that it has the potential for meniscus replacement. Our previous study has confirmed that autologous tendon grafts for acetabular labral reconstruction can fully or partially repair labral defects and convert to fibrocartilage-like tissue, indicating the fibrochondrogenic metaplasticity potential of tendon tissue^16^. The middle third of the patellar tendon autografts, fresh-frozen Achilles tendon allografts and Achilles tendon autografts were used in partial or total meniscus reconstruction in previous studies, but tendon graft alone without additional modification were reported not satisfactory in meniscus reconstruction^17–20^. Considering the distinct properties of tendon grafts, hamstring (semitendinosus and gracilis) tendons were not only reported to have significantly more density of collagen fibrils and cells than patellar tendon^21^, but also showed less donor site morbidity, decreased operative time, faster tendon-to-bone healing and regeneration potential after anterior cruciate ligament (ACL) reconstruction^22–28^. We therefore deduced that it might be a better choice for meniscus reconstruction. In this study, we transplanted a fresh semitendinosus tendon autografts in a rabbit model of total medial meniscectomy to investigate fibrochondrogenic metaplasticity potential and chondroprotective effect of the graft.

Meniscus were surrounded by synovial layer inside the articular capsule. Synovial coverage from the host plays a critical role in meniscus regeneration. Endogenous synovial-derived mesenchymal stem cells (SMSCs) are important cell sources for meniscus regeneration *in vivo* for its remarkable proliferation ability and chondrogenic potential^20^. Besides, Tendon-derived stem cells (TDSCs) are a subpopulation of residing stem cells within intact tendon tissue, with the capacities of self-renewal, clonogenicity, and three-lineage differentiation. Recently, Asai *et al*.^29^ demonstrated that TDSCs showed connective tissue progenitor properties and exhibited stronger chondrogenic ability than bone marrow stromal cells even in the absence of cytokines. These findings indicate that TDSCs in semitendinosus tendon are potential cell sources for meniscus regeneration *in vivo*. However, no study has evaluated the fibrochondrogenesis ability of SMSCs and TDSCs on tendon scaffolds until now. In this study, we produced decellularized semitendinosus tendon (DST) scaffolds with natural nanofibrous structures to mimic the microenvironment of tendon grafts, and evaluated the cell viability, proliferation, morphology and fibrochondrogenic differentiation capacities of SMSCs and semitendinosus TDSCs in DST scaffolds *in vitro*.

In the present study, we aim to confirm the potential of the application of semitendinosus tendon autograft in meniscus reconstruction. To achieve this purpose, we investigated cell viability, proliferation, morphology and the expression of fibrochondrogenic markers of SMSCs and semitendinosus TDSCs in DST scaffolds *in vitro*, and performed medial meniscus reconstruction by implanting a semitendinosus tendon autograft in a rabbit model of total medial meniscectomy to investigate the histologic and biomechanical properties of the grafts, as well as its protective effect for condylar cartilage from degenerative changes *in vivo*.

## Results

### Characterization of the DSTs

**Figure 1:**
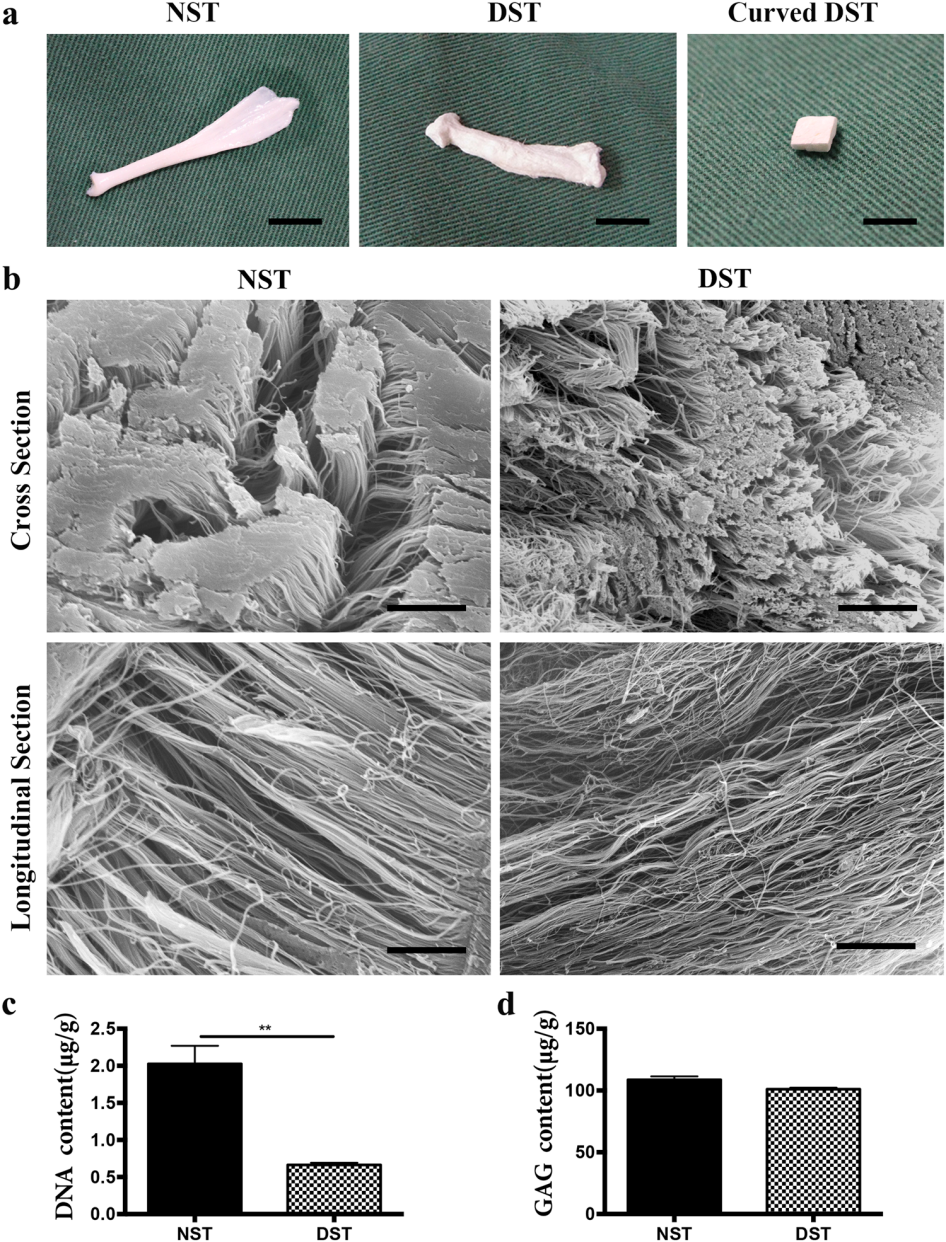
Characterization of the DST. (**a**) Gross appearance of tendon scaffolds. Scale bar = 5 mm. (**b**) SEM characterization for the surface topography of NST and DST. Scale bar = 10 μm. (**c**) DNA content of NST and DST. (**d**) GAG content of NST and DST.

To mimic the microenvironment of tendon grafts *in vivo*, we produced DSTs as described above. High magnification SEM micrographs showed the arrangement order and orientation of collagen fibrils in DSTs were well preserved (Fig. 1b). The DNA content of DSTs was significantly lower than that of native semitendinosus tendons (NSTs) (Fig. 1c), whereas the glycosaminoglycan (GAG) content of DSTs has no significant difference compared to NSTs (Fig. 1d). There was no difference of hardness, elastic modulus, elongation at break and degradation ratio between NSTs and DSTs (Supplementary Fig. S1a–c,e). The porosity of DSTs was significantly higher than that of NSTs, which may be caused by decellularization (Supplementary Fig. S1d). These results indicated that DSTs we produced were cell-free, possessed similar extracellular matrix components with complicated three-dimensional microstructure and similar physical-mechanical characteristics with natural tendon tissue, which could successfully mimic the microenvironment of tendon grafts *in vivo*.

### Cell viability, distribution, proliferative ability and morphology in DSTs

Since SMSCs were main endogenous cell sources for meniscus regeneration, and TDSCs were residing stem cells within the intact tendon tissues, we isolated both types of stem cells to evaluate the fibrochondrogenesis capability of SMSCs and TDSCs on tendon scaffold. After expansion, the rabbit SMSCs and TDSCs were presented as spindle or fusiform in cell colonies at passage 0 and changed into homogeneous spindle at following passages. The rabbit SMSCs and TDSCs successfully differentiated toward osteogenesis, adipogenesis, and chondrogenesis after induction, as identified by alizarin red, oil-red O and toluidine blue staining respectively (Supplementary Fig. S2).

**Figure 2:**
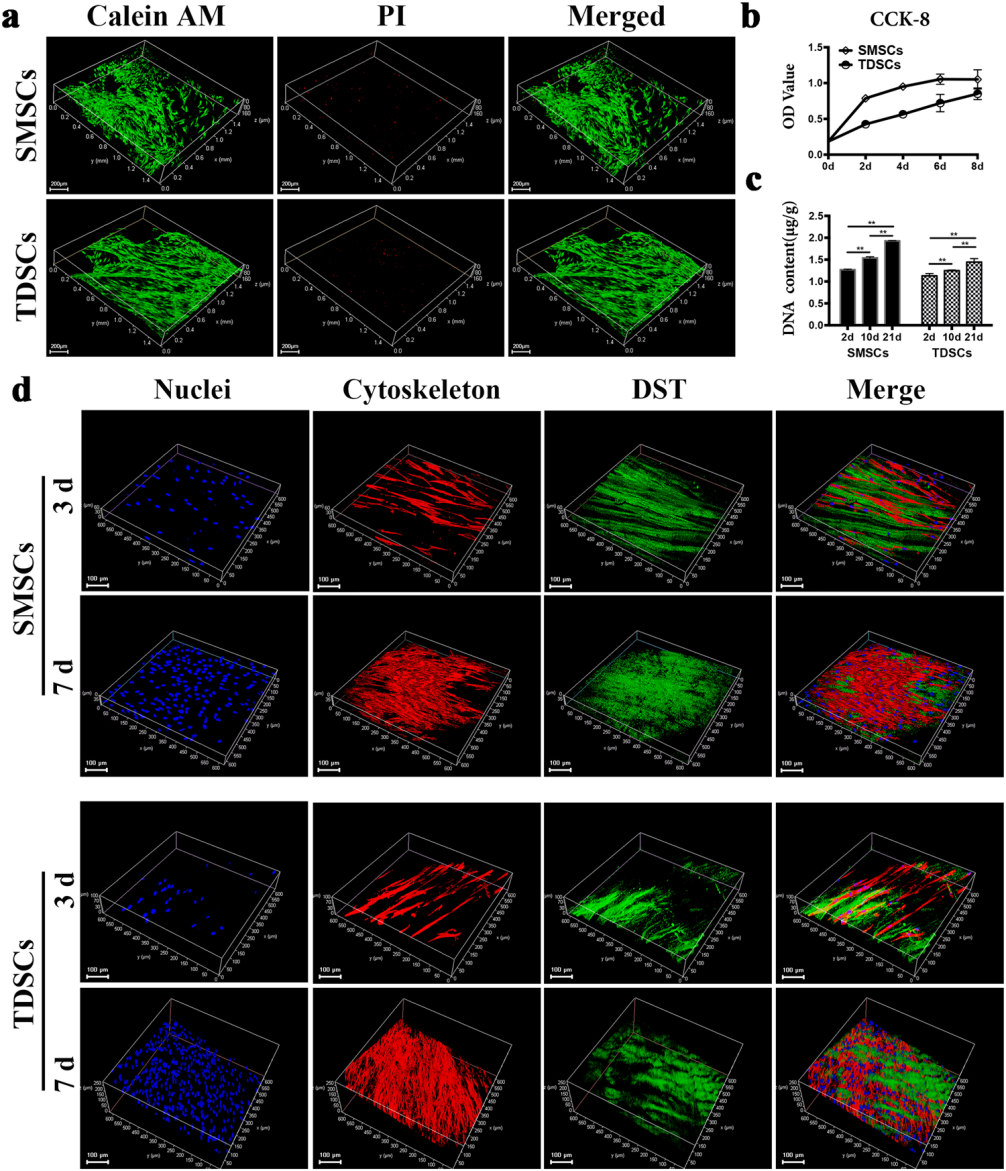
Viability, proliferation and morphology of SMSCs and TDSCs in DSTs *in vitro*. (**a**) LIVE/DEAD staining analysis of SMSCs and TDSCs seeded in DSTs after 7 days by fluorescence microscopy, with live cells stained green (calcein AM) and dead cells stained red (PI). Scale bar = 200 μm. (**b**) OD values of CCK-8 assay of SMSCs and TDSCs cultured in DSTs (n = 3). (**c**) DNA content of SMSCs and TDSCs seeded in DSTs at day 2, 10 and 21 (n = 3, **p < 0.01). (**d**) Cell morphology of SMSCs and TDSCs seeded in DSTs by fluorescence microscopy, with nuclei stained blue, cytoskeleton stained red and DSTs displayed green. Scale bar = 100 μm.

To detect the bioactivity of SMSCs and TDSCs in the DSTs, live/dead assay was performed after 7 days of culture. Results showed that both SMSC and TDSCs were evenly distributed and remained viable in DSTs. Few cells were observed dead (Fig. 2a). This indicated that DSTs could support cell retention and activity of both TDSCs and SMSCs.

The proliferative ability of TDSCs and SMSCs was detected by Cell Counting Kit-8 test. The TDSCs and SMSCs in DSTs displayed the same increasing proliferative tendency during the culture period of 1–7 days (Fig. 2b). Similar to the CCK-8 results, assessment of DNA content showed that DNA contents in DSTs at day 10 and 21 was significantly increased compared with that at day 2 (n = 3, *p < 0.05 versus day 2) (Fig. 2c). These results suggested that DSTs could promote the proliferation of TDSCs and SMSCs.

To evaluate cellular morphology of TDSCs and SMSCs in DSTs, cytoskeleton immunostaining was performed. Figure 2D showed the representative images of cellular morphology after 3 and 7 days of culture in DSTs with fibrochondrogenesis treatment. After 3 days’ culture, TDSCs and SMSCs were well attached on the surface of the scaffold, and showed a tendency of arrangement along the orientation of collagenous fibers of the scaffold. At day 7, a denser cell cluster was observed, suggesting significant cell proliferation in DSTs. SMSCs and TDSCs aligned in accordance with the direction of collagen fibrils.

### Fibrochondrogenic differentiation of TDSCs and SMSCs in DSTs

**Figure 3:**
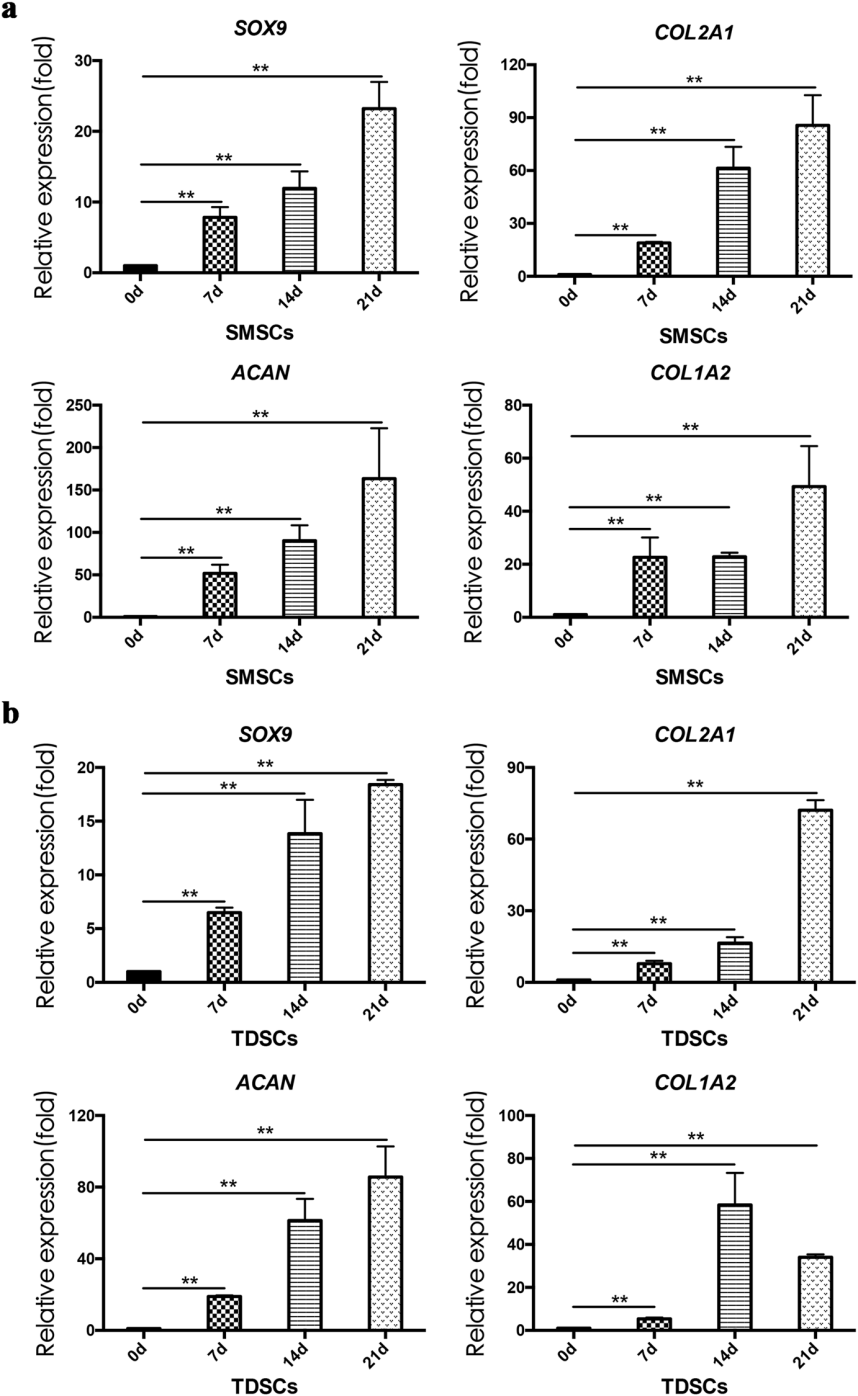
The expression of fibrochondrocyte-specific genes SOX9, COL2A1, ACAN and COL1A2 of SMSCs and TDSCs in DSTs *in vitro*. (**a**) SMSCs. (**b**) TDSCs. (n = 3, **p < 0.01).

We then further evaluated the capability of fibrochondrogenic differentiation of SMSCs and TDSCs in DSTs by examining the mRNA expression level of fibrochondrogenic-specific markers: SOX9, a key transcription factor during fibrochondrogenic differentiation process; Col1A2, Col2A1 and aggrecan (ACAN), the major extracellular matrix proteins. The initial relative mRNA expression levels of all four fibrochondrogenic genes between native tendon, native meniscus, SMSCs and TDSCs were exhibited at Supplementary Fig. S3. During 3 weeks of differentiation induction *in vitro*, the mRNA expression levels of all four fibrochondrogenic genes were upregulated over time in both TDSCs and SMSCs in DSTs (Fig. 3). The ratio between collagen II to collagen I mRNA level of native meniscus, native tendon, SMSCs and TDSCs was exhibited at Supplementary Fig. S4. These results indicated the superior fibrochondrogenic differentiation capacity of both TDSCs and SMSCs in DSTs, which are provided for next *in vivo* validation.

### General evaluation of implants

**Figure 4:**
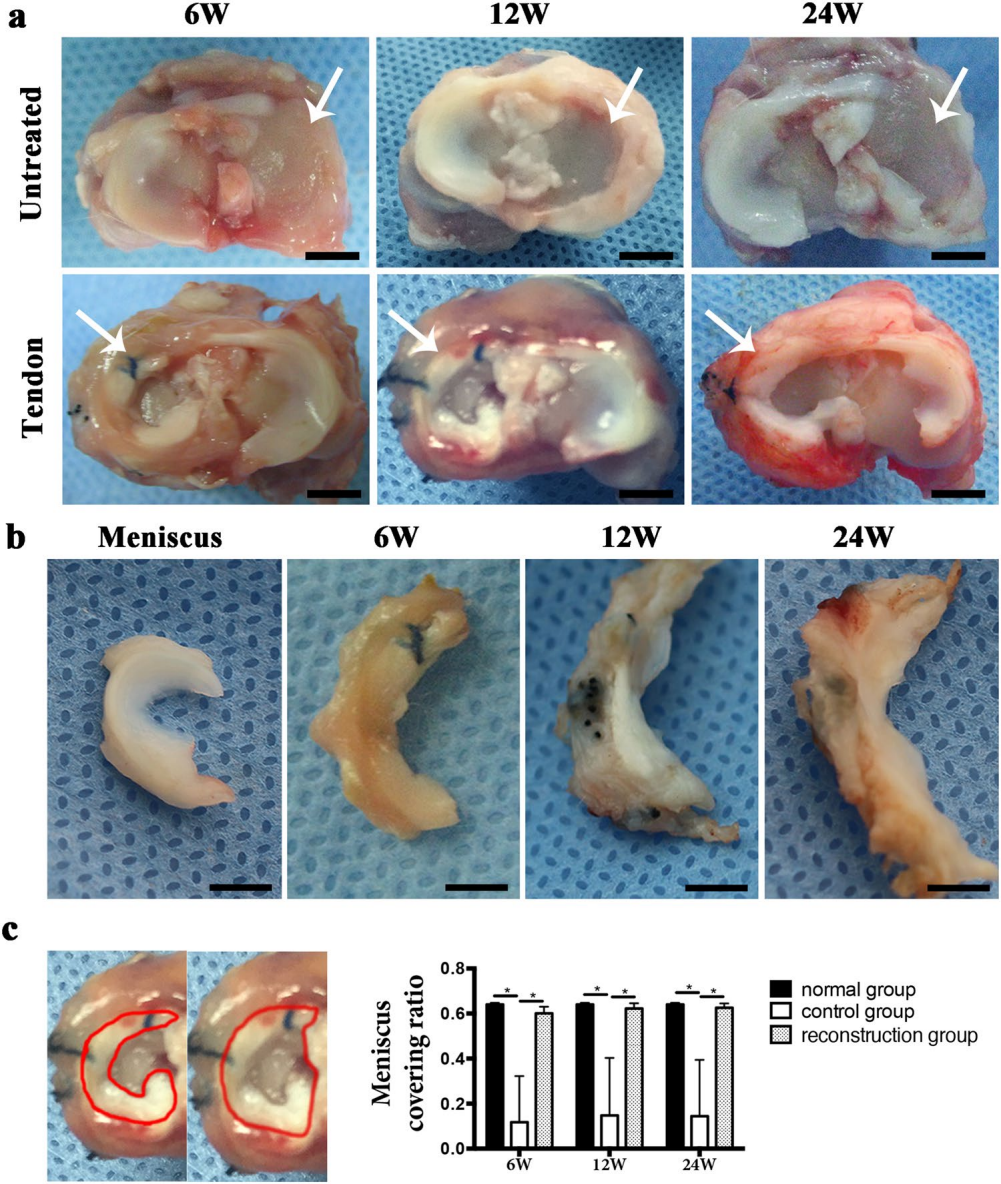
(**a**) Gross observation of regenerated meniscus on tibial plateau at 6, 12 and 24 weeks post-operative of untreated group and transplantation group. Scale bar = 5 mm. (white arrow, medial tibial plateau). (**b**) Gross observation of native meniscus and regenerated meniscus at 6, 12 and 24 weeks post-operation of transplantation group. Scale bar = 5 mm. (**c**) Area ratio of the medial meniscus to the whole medial tibial plateau in normal group, control group and transplantation group. (n = 5, *p < 0.05).

To verify the potential of semitendinosus tendon autografts in meniscus reconstruction, meniscus reconstruction with semitendinosus tendon autografts was performed on the right legs of total medial meniscectomy rabbit model, while left legs of the rabbit model without transplantation were served as a control. All rabbits recovered well from the operation without complications. Macroscopically, tendon grafts were intact and well attached to joint capsule in crescent-shape in the experimental group. No regenerated meniscus was seen in most of the control group with total meniscectomy without meniscus horn remnant, very few of them presented synovium meniscus due to the meniscus horn remnant (Fig. 4a and b). The area ratio of medial meniscus to the whole medial tibial plateau in experimental group increased more closely to normal meniscus over time, there was no significant difference between experimental group and normal control group. Whereas in meniscectomy group, the ratio was much lower than that of transplantation and normal control group (Fig. 4c).

### Histological assessment of implants

**Figure 5:**
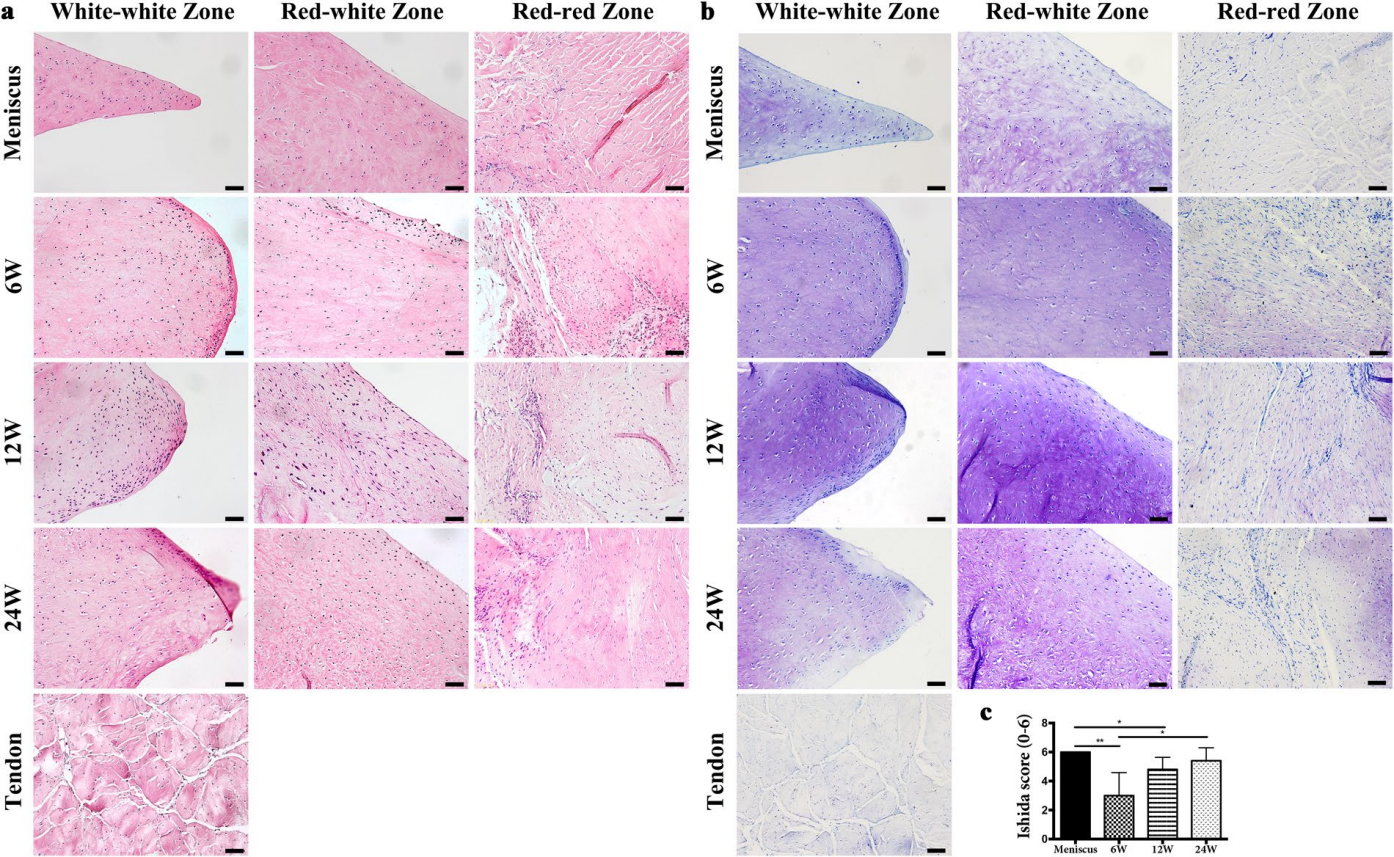
H&E staining, toluidine blue staining and Ishida score of native meniscus, native tendon and regenerated meniscus at 6, 12 and 24 weeks post-operative of transplantation group in white-white zone, red-white zone and red-red zone. Scale bar = 50 μm. (**a**) HE staining. (**b**) Toluidine blue staining. (**c**) Ishida score.

Hematoxylin and eosin (H&E) staining was performed to investigate the histological change of the tendon grafts (Fig. 5a). In white-white zone and red-white zone, the native meniscus consists of vast extracellular matrix with a relatively sparse cell population, fibrochondrocytes are situated in well-defined lacunae either separately or paired. Blood vessels were only observed in red-red zone. While in native tendon, tendocytes and vessels were aligned among collagen fiber bundles. At week 6 post-operation, synovium infiltration and angiogenesis was observed in red-red zone in all the grafts. Synovial tissue also covered the graft surfaces. Within the graft, some of the grafts showed cell proliferation and fibrochondrogenesis with homogeneous ECM (Fig. 5a), whereas some showed loose arrangement of collagen fibers and parallel arrangement of the spindle-shaped fibroblast cells in white-white zone and red-white zone, which was more similar to the native tendon (Supplementary Fig. S5). At 12 weeks, the grafts presented hypercellularity compared with the native meniscus. Oval- or round-shaped fibrochondrocytes appeared in white-white zone and red-white zone of the grafts. At 24 weeks, cell number within the grafts decreased to the level of the native meniscus. Homogeneous and dense extracellular matrix (ECM) was observed, similar to that of normal meniscus. Most cells in white-white zone and white-red zone turned into fibrochondrocyte phenotype, while cells in red-red zone were mixture of spindle-shaped fibroblast cells and fibrochondrocytes, which was in accordance with the native meniscus.

Toluidine blue (TB) staining was performed to detect proteoglycan formation of the implants. At 6 weeks, some of the grafts with cell proliferation and fibrochondrogenesis showed moderate staining of TB (Fig. 5b), while some of the tendon grafts were barely stained in white-white zone and red-white zone, which corresponded to the native tendon (Supplementary Fig. S5). Slight staining of proteoglycan was observed in all the grafts in the red-red zone, which suggested that fibrochondrogenesis first took place in the red-red zone due to angiogenesis and cell infiltration. At 12 weeks, the implants exhibited strong TB staining in white-white zone and red-white zone, suggesting extraordinary proteoglycan synthesis accompanied with cell proliferation. At 24 weeks, TB staining was positive but not as strong as that of 12 weeks, which suggested the proteoglycan synthesis of the implants returned to normal level and reached an equilibrium state similar to native meniscus. Ishida score of the regenerated meniscus improved over time, and at 24 weeks, there was no significant difference between the regenerated meniscus and native meniscus (Fig. 5c).

**Figure 6:**
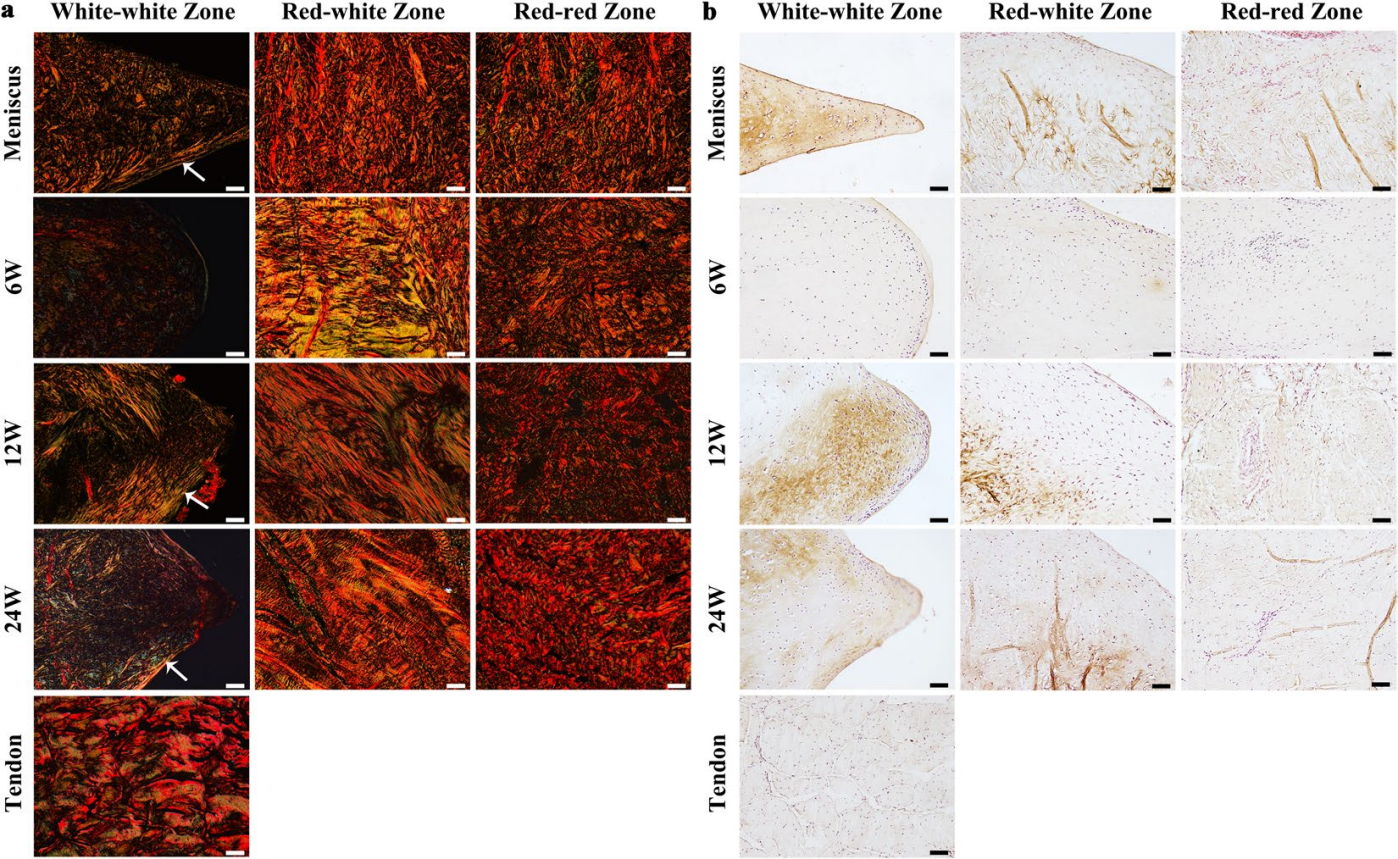
Picrosirius red and immunohistochemistry staining of collagen II of native meniscus, native tendon and regenerated meniscus at 6, 12 and 24 weeks post-operation of transplantation group in white-white zone, red-white zone and red-red zone. (**a**) Picrosirius red staining, with collagen type I stained red and collagen type III stained green. (white arrow, Radial collagen I fibers). Scale bar = 100 μm. (**b**) Immunohistochemistry staining of collagen II. Scale bar = 50 μm.

Picrosirius red staining (Fig. 6a) and immunohistochemistry of collagen II (Fig. 6b) were carried out to evaluate the amount of collagen component within the implants. Our results showed that native meniscus exhibited strong staining for collagen I in red-red zone and red-white zone, and moderate staining for collagen I and collagen II in white-white zone. Radial collagen I presented at the surface of native meniscus act as “tie fibres” to reinforce the meniscus (Fig. 6a, white arrow). The native tendon showed intense staining for collagen I and weak staining for collagen III but barely staining for collagen II. Similar to the native tendon tissue, at 6 weeks, the tendon grafts exhibited intense red staining for collagen I and weak green staining for collagen III in red-white zone and white-white zone. While in red-red zone, intense staining for collagen I and barely staining for collagen III was observed, indicated that collagen remodeling was first appeared at this region. At 12 and 24 weeks, radial collagen I fibers formulated at the surface of the regenerated meniscus, which was similar to the native meniscus (Fig. 6a, white arrow). The implants showed intense staining for collagen I in red-red zone and red-white zone, and moderate staining for collagen II in white-white zone, which indicated that further tissue remodeling took place in the red-white zone and white-white zone.

### Biomechanical properties of the implants

**Figure 7:**
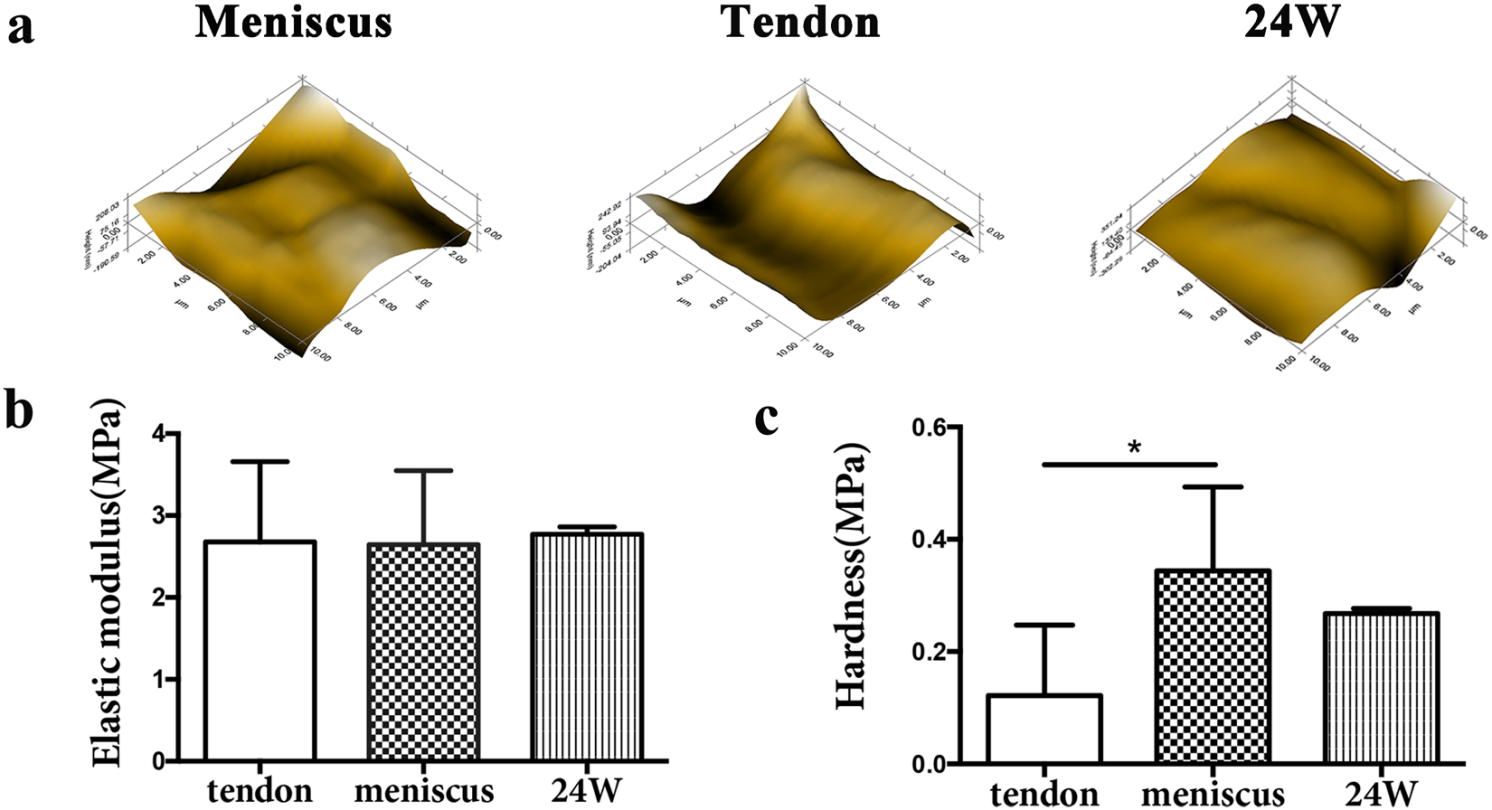
Biomechanical tests of native meniscus, native tendon and regenerated meniscus at 24 weeks post-operative of transplantation group. (**a**) Microscopic geomorphology of the surface of tendon and the femoral condylar contact surface of meniscus and regenerated meniscus was acquired during nanoindentation. (**b**) and (**c**) The biomechanical properties of each tissue were calculated with the biomechanical curves: (**b**) Elastic modulus; (**c**) Hardness (n = 5, *p < 0.05).

At 24 weeks post-surgery, we detected the biomechanical properties of the implants using nanoindentation. According to micro-scanning, the surfaces of native tendon and regenerated meniscus were as smooth as native meniscus (Fig. 7a). With regard to elastic moduli, there were no significant differences between native tendon, native meniscus and implants at 24 weeks (Fig. 7b). As for the hardness, native meniscus was significantly harder than native tendon, while there was no significant difference between implants at 24 weeks and native meniscus (Fig. 7c). Therefore, biomechanical testing indicated that mechanical strength of the regenerated meniscus at 24 weeks was similar with native meniscus.

### Evaluation of chondroprotective effect of the meniscus reconstruction

**Figure 8:**
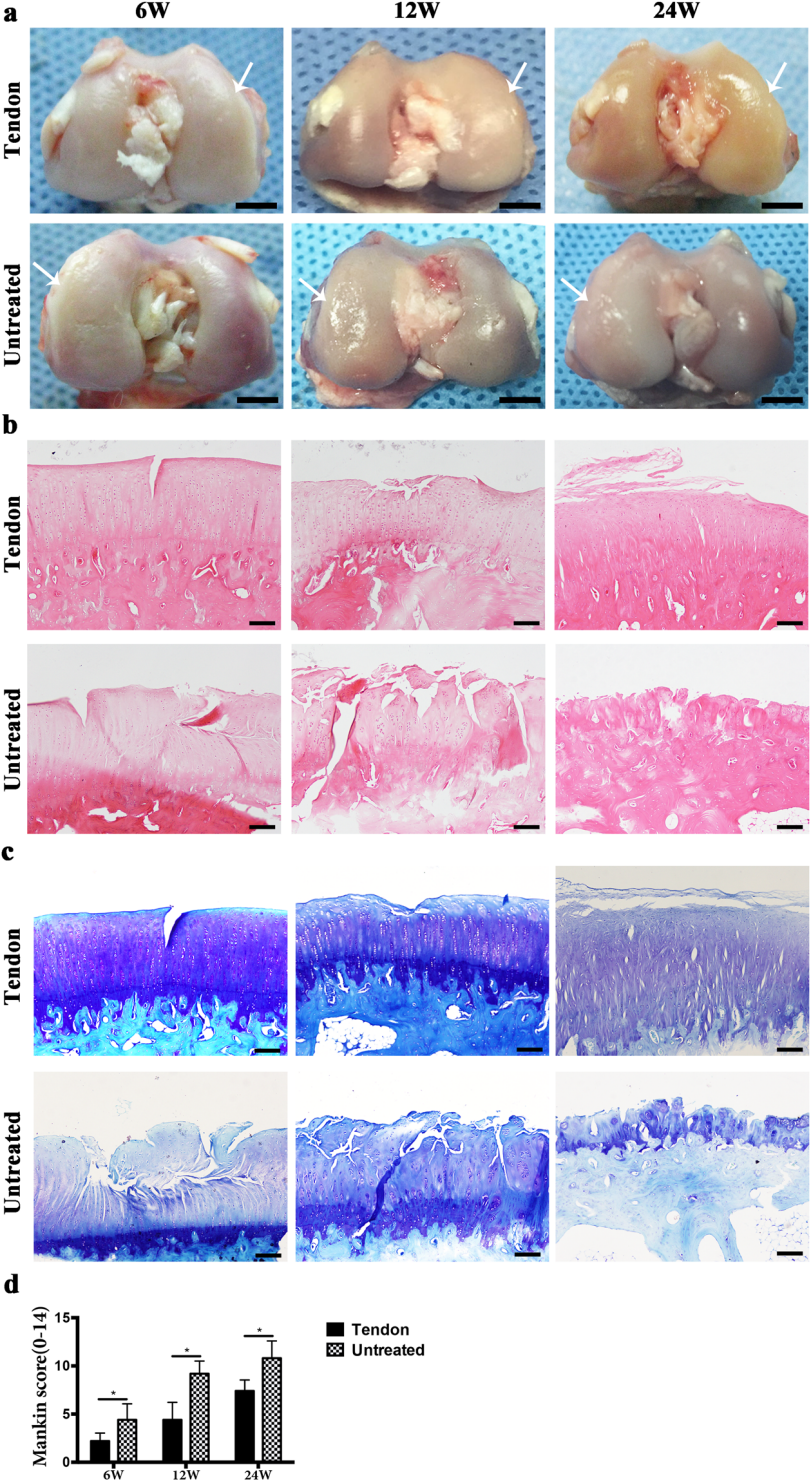
Macroscopic and histological analyses for articular cartilage at the medial femoral condyle. (**a**) Macroscopic features of femoral condyle cartilage. Arrows indicate medial femoral condyle. Scale bar = 5 mm. (**b**) HE staining of medial femoral condyle cartilage. Scale bar = 100 μm. (**c**) TB staining of medial femoral condylar cartilage. Scale bar = 100 μm. (**d**) Mankin score. (n = 5, *p < 0.05).

As for the femoral condylar cartilage, macroscopically, moderate cartilage erosion was observed at 6 weeks and progressed over 12 and 24 weeks in the untreated group. In the transplantation group, cartilage erosion was slight at 6 weeks and developed slowly, better preserved at 12 and 24 weeks compared with the control group (Fig. 8a). Histologically, H&E staining and toluidine blue staining also showed similar results with macroscopic findings: Cartilage degeneration progressed rapidly in the untreated group, whereas cartilage was significantly better preserved in transplantation group (Fig. 8b and c). Mankin scores in transplantation group were significantly lower than those of untreated group (Fig. 8d).

## Discussion

In this study, we confirmed that DSTs were capable to support cellular viability, distribution, proliferation and fibro-chondrogenic differentiation of both TDSCs and SMSCs *in vitro*. Transplantation of semitendinosus tendon autograft in rabbit model resulted in a meniscus substitution, of which the cover area, cellular population, collagen type, proteoglycan content, and biomechanical properties were similar to that of normal meniscus, demonstrating that semitendinosus tendon autograft is a promising alternative substitution in meniscus reconstruction, provided a clinically feasible treatment option.

Compared with synthetic material scaffold and allografts, autografts remain to be the standard for its excellent biocompatibility without toxicity, immunogenicity and risk of pathophoresis. Tendon grafts have been used in partial or total meniscus reconstruction, and showed different degrees of cartilage protective effects and grafts remodeling outcome due to their distinct properties and operation procedures. According to previous studies, patellar tendon or Achilles tendon alone only ended up with an inferior substitution in meniscus reconstruction^17–20, 30^. Semitendinosus tendon autograft has been reported with less donor site morbidity and faster quadriceps recovery in its wide application in ACL reconstruction^31, 32^. Higher density of cells and collagen fibrils in semitendinosus tendon than bone-patellar tendon-bone autografts have also been reported^21^. Considering that graft remodeling and regeneration of the tissue was influenced by stem cell density and scaffold structure, we used fresh semitendinosus tendon autografts for rabbit meniscus reconstruction in this study. Our findings showed that regenerated meniscus substitution with fibrocartilage-like properties and chondroprotective effect were able to be achieved by using a fresh semitendinosus tendon autograft.

In our meniscus reconstruction model, endogenous SMSCs and TDSCs from semitendinosus tendon autografts were regarded as two most important stem cell sources. Previous studies showed host synovium tissue coverage on the grafts after transplantation in a meniscus reconstruction model, which supported the hypothesis of meniscus regeneration by endogenous SMSCs^19^. In our study, synovial coverage on the surface and integration site of tendon grafts was observed at 6 weeks post-surgery (Fig. 5). Furthermore, previous studies suggested that TDSCs inside the tendon was also a potential cell source for meniscus regeneration according to Ozeki *et al*., whose studies demonstrated that original cells in tendon graft were retained after meniscus reconstruction and probably contributed to meniscal regeneration^19, 20^. In this study, we isolated SMSCs from rabbit knee synovium and TDSCs from semitendinosus tendon, and verified three lineage differentiation ability of SMSCs and TDSCs, confirming that both SMSCs and TDSCs were available cell sources in our meniscus reconstruction model.

Microenvironment of the scaffolds can influence the effect of transplantation by affecting cell behavior. Decellularized extracellular matrix can well preserve the complex composition of original ECM composition, multiple bioactive factors and microstructure of the natural materials, and thus it was extensively used in tissue engineering. DSTs we developed preserved the parallel type I collagen fibers and proteoglycan, which were the ideal representative of the *in vivo* microenvironment of tendon graft (Fig. 5). Lots of researches have shown that type I collagen scaffold is a kind of natural biological material with the ability of inducing stem cells to differentiate into cartilage^33, 34^. Well-aligned structures of the abundant type I collagen nanofibers in DSTs were similar with the collagen arrangement in red-red zone and red-white zone of native meniscus, providing a specific microenvironment and elastic modulus for meniscus tissue engineering. Previously, decellularized tendon tissues have been revealed to have the capacity of supporting stem cells distribution, alignment, proliferation and tenogenic or osteogenic differentiation *in vitro* ^35, 36^. Nevertheless, no studies have validated the capacity of stem cells in chondrogenic differentiation of stems cells in tendon ECM scaffolds to date. In this study, we verified that DSTs were able to support SMSCs and TDSCs distribution, proliferation and fibrochondrogenesis differentiation *in vitro*. SMSCs and TDSCs expressed high levels of fibrochondrogenesis markers SOX9, COL1A2, ACAN and COL2A1 in DSTs, suggesting that the microenvironment of the semitendinosus tendon scaffold was suitable for meniscus regeneration.

Meniscus is a heterogeneous tissue, in which vascularization and fibroblast-like cells primarily presents in the red-red zone and red-white zone with abundant collagen type I and sulfate glycosaminoglycans (GAGs), rather than white-white zone, which was nonvascular and primarily consists of hyaline-like chondrocyte, GAGs and collagen type II. In ACL reconstruction, three consecutive distinguishable phases of postoperative remodeling could be identified for the tendon grafts, which were called “ligamenlization”^37^. In this study, we observed similar “meniscusization” process. In the early stage of 6 weeks, hypocellularity was found in white-white zone and red-white zone in some of the grafts (Supplementary Fig. S5). The red-red zone of the grafts was sutured to the peripheral synovium and joint capsule, which was first infiltrated with synovium tissues and vascularized. Collagen type III in tendon tissue diminished and collagen type I increased in abundance in this region at early stage. In 12 weeks, an increase of cell density accompanied with ECM expansion prompted structural remodeling of the regenerated grafts. Proteoglycan formation and collagen remodeling was observed to be progressing drastically at this stage. In addition to type I collagen, a small amount of type II collagen synthesized in white-white zone presented heterogeneous tissue formation of the regenerated meniscus. In 24 weeks, the regenerated meniscus was similar to native meniscus in cell count, proteoglycan formation, collagen fiber distribution and biomechanical properties (Figs 5 and 6). All these results suggested that our tendon grafts ended up in a substitution similar to native meniscus. Previous study of Achilles tendon allograft for complete meniscus reconstruction showed areas of endochondral ossification inside the allograft^18^. In this study, we did not find endochondral ossification area inside the graft. The hardness of implants at 24 weeks improved significantly compared with the native tendon, which was an evidence of ECM accumulation.

When the knee joint is weight loaded, the tensile strength based on the circumferential collagen fibre bundles and the anatomical insertions of the anchoring horns of the meniscus counteracts extrusion of the meniscus^38^. Tensile property of the tendon grafts we used in this study is close to the native meniscus. In the early stage post-operation, tendon graft act as a substitute for meniscus in load transmission and shock absorption, therefore appropriate tensile properties can protect the graft from extrusion. In later remodeling stages, formation of collagen type II, proteoglycan and radial collagen type I “tie fibres” in the surface of the regenerated meniscus contributed to the hardness property of regenerated meniscus. This construction of hardness and tensile properties makes the regenerated meniscus a perfect shock absorber and load transmitter of the knee.

Surgical techniques are of vital importance to postoperative outcome and pathology prognosis^39^. In this study, by fixing two ends of the knitted tendon through bone tunnel on tibia and suturing the graft onto the joint capsule, we avoided graft displacement and tear after surgery. In a previous pilot study, of which four semitendinosus tendon autografts and one patellar tendon were used in human meniscus reconstruction, the authors fixed the anterior horn of the tendon by a bone tunnel and sutured the tendon body to the joint capsule, but for technical convenience, they sutured the posterior horn of the tendon to the posterior meniscal remnant rather than fixed through a bone tunnel. Fragmentation of most tendon grafts and poor fibrocartilage metaplasia was observed at 9 to 24 months post-surgery^30^. It is considerable that suturing the posterior horn of the tendon to the posterior meniscal remnant was not enough for fixation of the grafts. Hypermobility of the graft influenced the remodeling and fibrocartilage metaplasia of the graft. Fixation by bone tunnel at both ends of the tendon would achieve better anatomical fixation, and correspond more closely to the status of stress and load transmission of normal meniscus.

Unlimited weight-bearing activity was allowed for rabbits post-surgery in this study. However, in human meniscus transplantation, the implant should be protected for 4 to 6 weeks with joint immobilization postoperative^39^. Further research needs to be conducted to investigate the influence of postoperative rehabilitation program on the prognosis.

## Materials and Methods

All animal experiments were complied with the “Guide for the Care and Use of Laboratory Animals” published by the National Academy Press (NIH Publication No. 85e23, revised 1996) and approved by the Animal Care and Use Committee of Peking University. Skeletally mature Japanese big-ear rabbits weighing 3.0–3.5 kg were used in this study.

### Isolation and culture of rabbit TDSCs and SMSCs

Rabbit SMSCs and semitendinosus TDSCs were isolated and cultured as previously described with slight modification^40, 41^. After sacrifice, semitendinosus tendon was excised from both knees of rabbits for isolation of TDSCs. The tendon sheath and surrounding paratenon were removed, and tendon tissues were cut into small pieces and followed by digestion with 0.2% collagenase type I (Invitrogen, Carlsbad, CA, USA) in α-minimal essential medium (α-MEM, Gibco) for 1 h at 37 °C. The cells were then suspended in α-MEM supplemented with 10% fetal bovine serum (FBS), 100 U/mL penicillin, 2 mM L-glutamine (Invitrogen, Carlsbad, CA, USA) and 100 mg/mL streptomycin. For isolation of SMSCs, synovial tissues were harvested from rabbit knees and washed three times with PBS, then digested with 0.2% collagenase type I (Invitrogen, Carlsbad, CA, USA) in α-MEM for 30 min at 37 °C. The released cells were suspended in complete medium. Fresh medium was replaced every 3 days.

### Multi-differentiation assay

The multi-differentiation potential of the SMSCs and TDSCs was examined at passage 2. For osteogenesis, TDSCs and SMSCs (8 × 10^3^ cells/cm^2^) were cultured in rabbit MSC osteogenic differentiation medium (RBXMX-90021; Cyagen Biosciences Inc.). After 21 days of culture, cells were fixed with 4% formalin for 30 min, washed twice with PBS and stained with 0.1% Alizarin red solution for 3–5 min. For adipogenesis, TDSCs and SMSCs (8 × 10^3^ cells/cm^2^) were cultured in rabbit mesenchymal stem cell adipogenic differentiation medium (RBXMX-90031; Cyagen Biosciences Inc.). After 14 days of culture, the cells were fixed with 4% formalin and stained with 0.5% fresh Oil-red O solution for 30 min. For chondrogenesis, a pellet culture system was used. About 2.5 × 10^5^ cells were pelleted into a micromass by centrifugation at 1000 rpm for 10 min in a 15-ml conical polypropylene tube and cultured in rabbit mesenchymal stem cell chondrogenic differentiation medium (RBXMX-90041; Cyagen Biosciences Inc.). After 21 days of culture, pellets were fixed with 4% paraformaldehyde for 30 min and then embedded in paraffin, cut into 5 μm thick sections, and stained with alcian blue solution for 30 min.

### DSTs preparation

NSTs were harvested from adult Japanese big-ear rabbits weighing 3.0–3.5 kg (Fig. 1a). After washing three times with PBS, tendon tissues were freezed by liquid nitrogen for 2 min and then thawed in saline solution at 37 °C for 10 min for five times repetitive. Then, tendon tissues were digested with trypsin enzyme at 37 °C for 2 h. Following three times washing in PBS for 30 min, tendon tissues were incubated in 1% triton for 12 h and then rotated in 5% SDS for 2 h. Finally, tendon tissues were washed five times in PBS with gentle rotation for 30 min. Then, the prepared DSTs were lyophilized and cut into 3mm × 3mm × 2mm pieces (Fig. 1a). For sterilization, 24 h of cobalt-60 irradiation were used.

### SEM characterization of DSTs

For topography characterization, NSTs and DSTs samples (n = 3 for each) were fixed in 2.5% glutaraldehyde for 2 h at 4 °C, and then conducted to critical point drying and gold sputter coating. A JEOL JSM-5600LV scanning electron microscopy at an accelerating voltage of 29 kV was used for observation.

### DNA and GAG content evaluation

For DNA and GAG content evaluation of the DSTs and NSTs, a Varioskan Flash reader (Thermo Scientific, Wyman Street Waltham, MA, USA) was used. Scaffolds were weighed and digested in a pre-prepared papain solution (Sigma) at 60 °C overnight. For measuring the content of DNA, 20 μL of digest solution was incubated at 37 °C for 1 h with 200 μL Hoechst 33258 working solution (2 μg/mL) (Polysciences Inc., Warrington, PA, USA). Then fluorescence intensities were measured at 360 nm for excitation and 460 nm for emission. The DNA content was calculated according to a standard curve of calf thymus DNA (Sigma). Proteoglycan content was tested from the GAG content with a 1,9-dimethylmethylene blue (DMMB; Sigma) dye-binding assay. 200 μL of DMMB reagent was mixed with papain digestion solution, and absorbance was measured on a Varioskan Flash instrument at 525 nm. The GAG content was calculated according to a standard curve based on chondroitin-6-sulfate from shark (Sigma).

### Cell culture on the DST

To investigate cell behavior of TDSCs and SMSCs on DSTs, TDSCs and SMSCs at passage 4 were used for *in vitro* culture. DST scaffolds were incubated with 1 ml complete Minimum Essential Medium α (MEM- α) containing 2 × 10^6^ cells for 2 h. After that, the DST scaffolds with cells were transferred into new 12-well plates and cultured in fibrochondrogenic differentiation medium, which was mixed with bFGF 1 ng/μL (100-18B; Peprotech) and rabbit mesenchymal stem cell chondrogenic differentiation medium (RBXMX-90041; Cyagen Biosciences Inc.), at 37 °C in 5% CO_2_. The media was changed every two days.

### Cell viability of TDSCs and SMSCs on DSTs

The cell viability at day 7 was assessed qualitatively using a Live/Dead Viability/Cytotoxicity Kit (Invitrogen, USA). Briefly, the cells-seeded DSTs were washed with PBS for three times, and incubated with 2 µM calcein AM and 4 µM EthD-1 working solution for 15 min at 37 °C and washed again with PBS. A confocal laser scanning microscope (Leica TCS-SP8 confocal microscopy; Leica, Nussloch, Germany) was used to capture images of live (green) and dead (red) cells.

### Cell proliferation and matrix formation *in vitro*

To assess the proliferative ability of TDSCs and SMSCs on DST scaffolds *in vitro*, we used a Cell Counting Kit-8 assay (CCK-8; Dojindo Laboratories, Kamimashiki Gun, Kumamoto, Japan). In brief, 200 μL of CCK-8 solution was added to the medium of cells-seeded DSTs (n = 3) and incubated at 37 °C for 4 h. The OD value was then measured at 450 nm with a Varioskan Flash instrument.

For DNA content measurement, the DST scaffolds seeded with TDSCs and SMSCs at day 2, 10 and 21 (n = 3 for each group at each time point) were managed according to method mentioned above.

### Cell morphology and alignment

To observe cell morphology and alignment on DSTs, both DSTs with TDSCs and SMSCs after 3 days and 7 days culture, were washed three to five times with PBS, fixed with 4% paraformaldehyde, and then incubated with rhodamine phalloidin (160 nM; Cytoskeleton Inc., Denver, CO, USA) for 1 h at 37 °C to reveal the cytoskeleton of the cells. Hoechst 33258 (1:800; Fanbo, Beijing, China) was used as a counter-stain of nuclei for 20 min and then examined under a confocal microscope.

### Quantitative real-time PCR analysis

To evaluate the fibrochondrogenic differentiation capacity of both TDSCs and SMSCs in DSTs, TDSCs and SMSCs were cultured in the DSTs for 7, 14, and 21 days. Then the mRNA expression levels of fibrochondrogenic-specific marker genes in the cells were examined using quantitative real-time PCR. Native tendon and native meniscus tissue were harvested from rabbits. TDSCs and SMSCs in plate culture before fibrochondrogenesis induction were served as control. At each time point, samples (n = 3) were rinsed with PBS, snap-frozen and pulverized in liquid nitrogen. Total cellular RNA was extracted with TRIzol solution (Invitrogen, Carlsbad, CA, USA). A RevertAidTM First Strand cDNA synthesis kit (Fermentas, Lithuania, USA) was used to synthesize cDNA. An ABI step-one plus Real-Time PCR System (Carlsbad, CA, USA) with SYBR Green PCR Master Mix (Toyobo, Osaka, Japan) were used for qPCR analysis. The primers for fibrochondrogenic-specific markers including SOX9, COL2A1, ACAN and COL1A2. GAPDH was served as the internal control. The sequences of the primers were shown in Supplementary Table 1. The relative gene expression levels were calculated by the ∆∆Ct method^42^. All experiments were performed in triplicate.

### Surgical procedures and postoperative treatment

Fifteen skeletally mature Japanese big-ear rabbits (total 30 knees) weighing 3.0–3.5 kg were included in this study, right knees received implant after meniscectomy while left knees only received meniscectomy. The rabbits were anesthetized intravenously with 10 mL ethylcarbamate (0.2 g/mL) during the operation. After skin disinfection and draping, an anteromedial parapatellar incision was made and the patella was everted. Total medial meniscectomies were performed on both right and left knees. Designed as control, the operatory of left knee was finished and the wound was closed. As to the right leg, the semitendinosus tendon was harvested through the same incision (Supplementary Fig. S6a), and the medial collateral ligament was cut off. The tendon was weaved using polyester knitted woven 3-0 (Supplementary Fig. S6b). Two 2.0 mm wide bone tunnels were made at insertion sites of the original medial meniscus on the tibia (Supplementary Fig. S6c). The weaved tendon was passed through the bony tunnel (Supplementary Fig. S6d). The peripheral area of the tendon was sutured to joint capsule with 4-0 sutures. The ends of the tendon were pulled tightly and tied. The medial collateral ligament was sutured and wound was closed in layers. The animals received antibiotic prophylaxis with 10 mg/kg penicillin for 3 days postoperatively. No joint immobilization method was used after surgery.

### Macroscopic observation of implants and femoral condylar cartilage

To evaluate the changes and chondroprotective effect of implants, rabbits were sacrificed at 6, 12 and 24 weeks after surgery, left knees and right knees were dissected. Macroscopic pictures of the meniscus and femoral condyles were taken, and the size of the regenerated meniscus was quantified using autoCAD software by measuring the area ratio of the medial meniscus to the whole area of the medial tibial plateau (Fig. 4c). The native meniscus, native semitendinosus tendon, tendon grafts of the reconstruction group and medial femoral condyles of both group were harvested for following analysis.

### Histological assessment

The native meniscus (n = 3), native semitendinosus tendons (n = 3), regenerated meniscus (n = 5) and the medial femoral condyles of each group (n = 5 per group) were fixed in 4% paraformaldehyde, the medial femoral condyles were then decalcified for 3 days. All the specimens were dehydrated in alcohol and then embedded in paraffin wax. The samples were sectioned in the coronal plane at 5 μm and stained with H&E and TB for proteoglycans. Histologic analysis of regenerated tissue and cartilage of the medial femoral condyles was performed with Ishida score and Mankin score, respectively. Native meniscus, native semitendinosus tendons and regenerated meniscus were also stained with picrosirius red for collagen type I and type III. Immunohistochemistry was performed with primary antibodies of rabbit anti-collagen type 2 (Calbiochem Cat No.: CP18-100UG; Novabiochem, Boston, MA, USA for collagen type II detection) at a dilution of 1:150.

### Nanoindentation assessment of repaired tissue

The biomechanical analysis of the tendon graft was performed using the nanoindentation. Samples (n = 5 at 24 weeks) were isolated from the anterior part of the tendon grafts. Native tendon (n = 5) and native meniscus samples (n = 3) were used as controls. Indentation assay was performed using the Tri-boIndenter (Hysitron Inc, Minneapolis, Minnesota, USA) with a 20 μm 90°conical probe tip. For each indentation, the maximum indentation depth was set to 500 nm, and the sample was loaded for the first 5 seconds once the tips contacted the surface of the samples, held at the maximum depth for 2 seconds, and unloaded in the last 5 seconds.

### Statistical analysis

Sample size was determined using power analysis (β = 0.1 and α = 0.05) with the difference of the two sample mean of 2.5 and standard deviation of 1 according to Mankin Score reported in previous studies^19^. The resulting sample size was 4, in case of unexpected lost, seventeen rabbits were finally included in this study. Two rabbits died after anesthesia were served as normal control group. Five rabbits per time point were killed. All the surgeries were done by an independent researcher to eliminate any bias. The investigator for Mankin Score evaluation was blinded. Ishida score evaluation was not blinded because of the apparent difference between regenerated meniscus and native meniscus. All the *in vitro* experiments were repeated at least three times. SPSS 18.0 statistical software was used for statistical analysis. Data were presented as mean ± SD. The statistical differences between groups were calculated using student’s t-test or ANOVA. P < 0.05 was considered significant.

Supplemental Information includes Supplemental Experimental Procedures, six figures, and one table can be found with this article.

## Electronic supplementary material

Supplementary Information
