# Supplementary material for: The potential of using semitendinosus tendon as autograft in rabbit meniscus reconstruction
Source: Sci Rep. 2017 Aug 1;7:7033. doi: 10.1038/s41598-017-07166-z (PMC5539314; doi:10.1038/s41598-017-07166-z)
Supplement: Supplementary file 1 — Supplementary Information [file 41598_2017_7166_MOESM1_ESM.doc]

**The potential of using semitendinosus tendon as autograft in rabbit meniscus reconstruction**

**Chenxi Li, Xiaoqing Hu, Qingyang Meng, Xin Zhang, Jingxian Zhu, Linghui Dai, Jin Cheng, Mingjin Zhong, Weili Shi, Bo Ren, Jiying Zhang, Xin Fu, Xiaoning Duan, Yingfang Ao** *

Institute of Sports Medicine, Beijing Key Laboratory of Sports Injuries, Peking University Third Hospital, 49 North Garden Road, Haidian District, Beijing 100191, People's Republic of China.

*corresponding.author: aoyingfang@163.com

**Supplementary Materials:**

**Supplementary methods:**

***Elongation at break***

The elongation at break of the DST, NST and native meniscus were tested using an AGS-X Precision Universal Tester (SHIMADZU, Japan) with an elongation rate of 15mm/min. For each group, five samples were tested.

***Degradation properties, water uptake and porosity***

To calculate the degradation rate, water uptake and porosity of DST and NST, dry weight of freeze-dried scaffolds was weighed (Wd). And then, the scaffolds were immersed in PBS (pH 7.4) at 37 ℃ for several days. The swollen scaffolds were weighed by a microbalance after removing the excess of water on the surfaces with filter papers. The weight after the first night was recorded as W_0_, while others were recorded as Wt. The degradation ratio of the scaffolds was calculated as [(W_0_-Wt)/W_0_] ×100%.

For water uptake experiment, the scaffolds were immersed in PBS for 3 days and weighed after removing the excess of water on the surfaces with filter papers (W_3_). The water uptake was calculated as [W_3_-Wd/ W_0_] ×100%. To calculate the porosity of the scaffolds, the pore volume of the scaffolds (Vp) was represented by the volume of water, which was calculated from the mass and density of the water. The mass of the water absorbed by the scaffolds was calculated by W_0_-Wd. The volume of the scaffolds (Vs) was calculated from height, width and depth. The porosity was calculated as Vp/Vs×100%.

***Nanoindentation assessment of repaired tissue***

The biomechanical analysis of the decellularized tendon graft was performed using the nanoindentation. Samples (n=5) were prepared as the methods described in 4.3. The native tendon samples (n = 5) were used as control. Indentation assay was performed as methods described in 4.14.

Results:


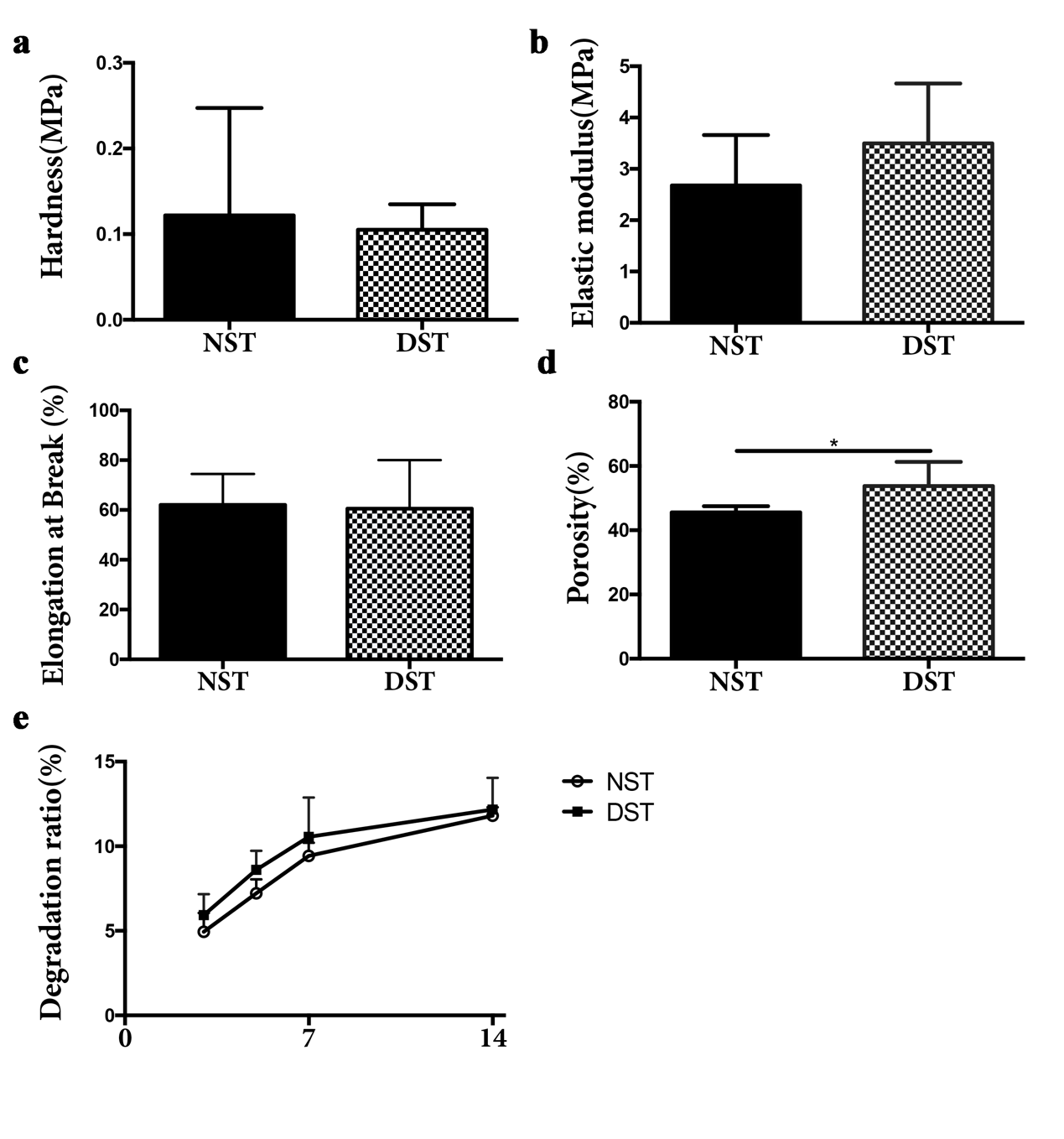


Supplementary Figure S1. Physical-mechanical characteristics of the DST and NST. (a), Hardness. (b), Elastic modulus. (c), Elongation at break. (d), Porosity. (e), Degradation ratio. (n=5, *p<0.05).


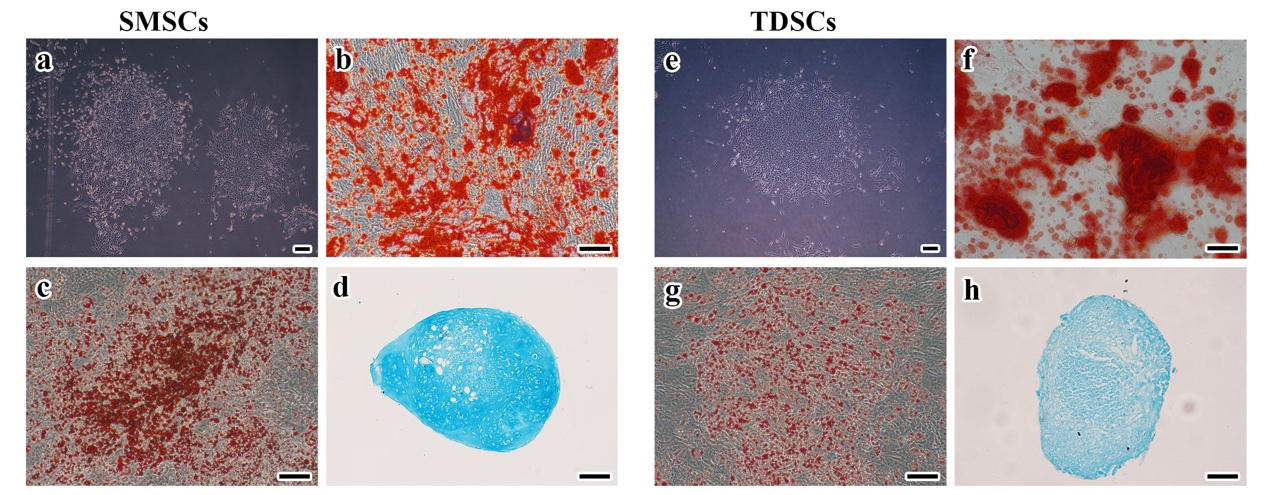


Supplementary Figure S2. Multiple-differentiation potentials of SMSCs and TDSCs . (a) and (e) Colonies were formed by SMSCs (a) and TDSCs (e) at P0. (b) and (f) Osteogenesis was examined using alizarin red staining. (c) and (g) Adipogenic capacity was identified with oil red O staining. (d) and (h), chondrogenic potential was verified by toluidine blue staining. Scale bar = 100μm.


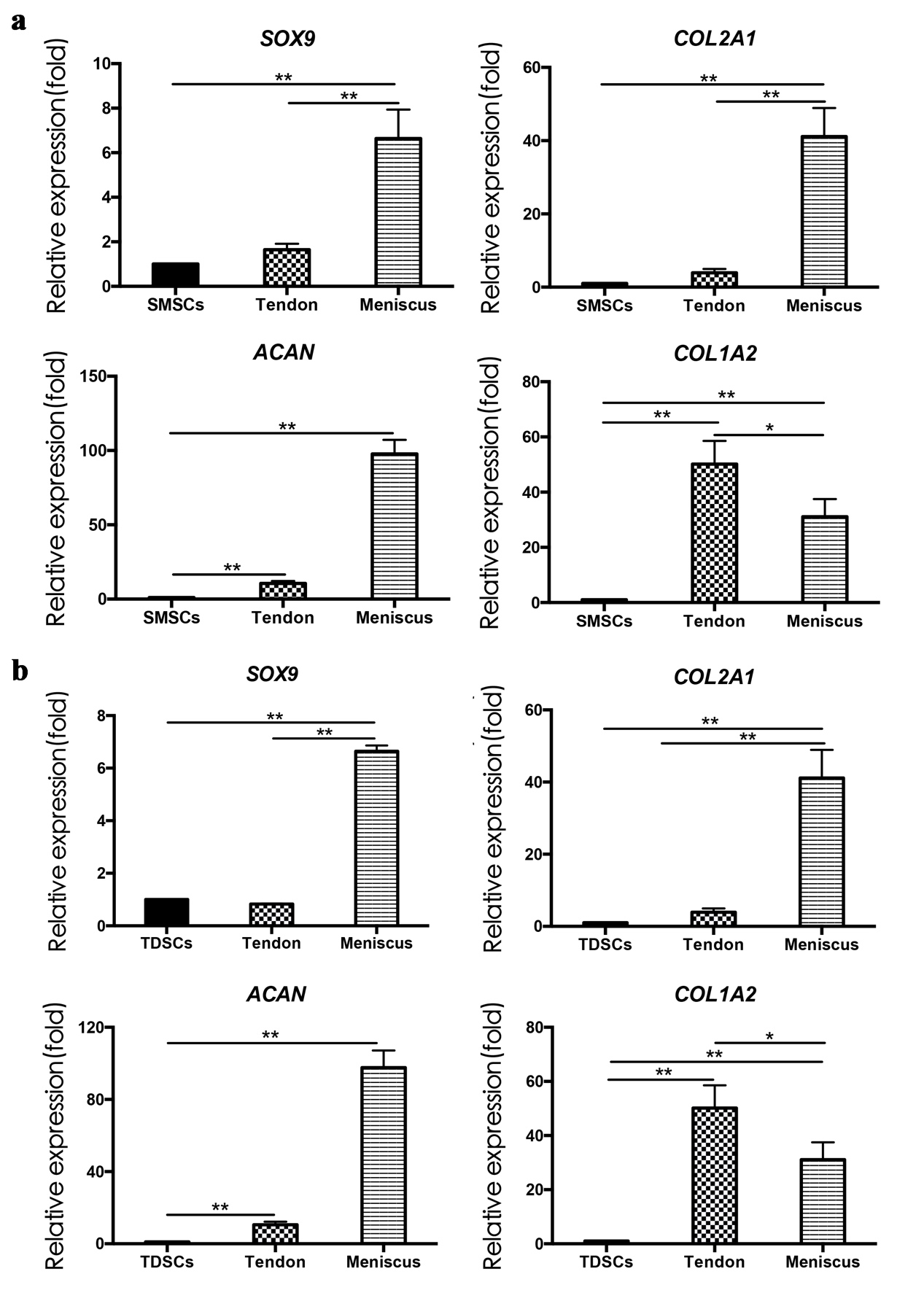


Supplementary Figure S3. The expression of fibrochondrocyte-specific genes SOX9, COL2A1, ACAN and COL1A2 of SMSCs, TDSCs, native tendon and native meniscus. (a), SMSCs. (b), TDSCs. (n=3, **p<0.01).


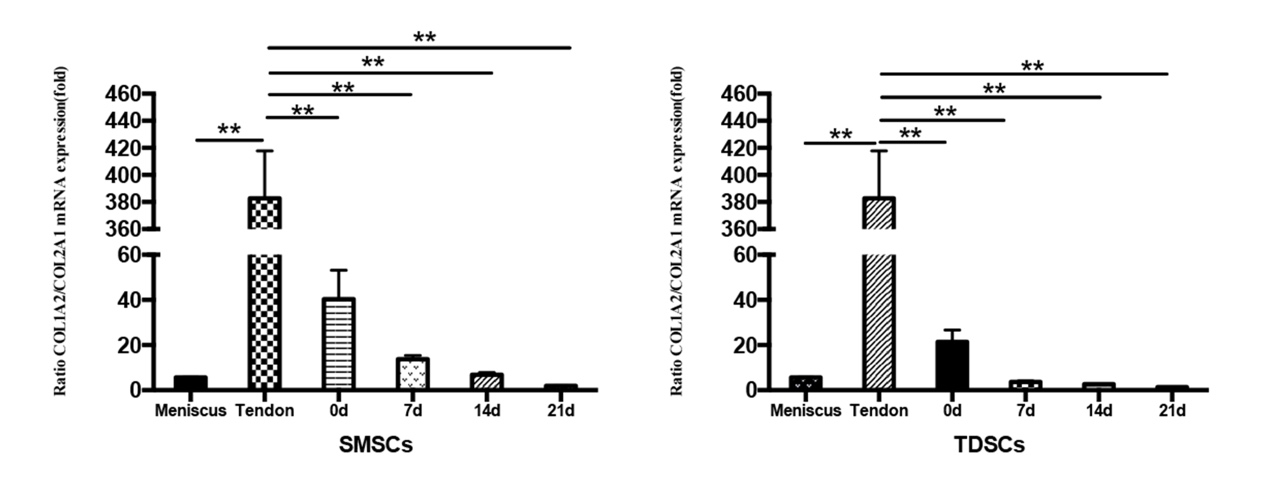


Supplementary Figure S4. Ratio between collagen II to collagen I mRNA level of native meniscus, native tendon, SMSCs and TDSCs. (a), SMSCs. (b), TDSCs. (n=3, **p<0.01).


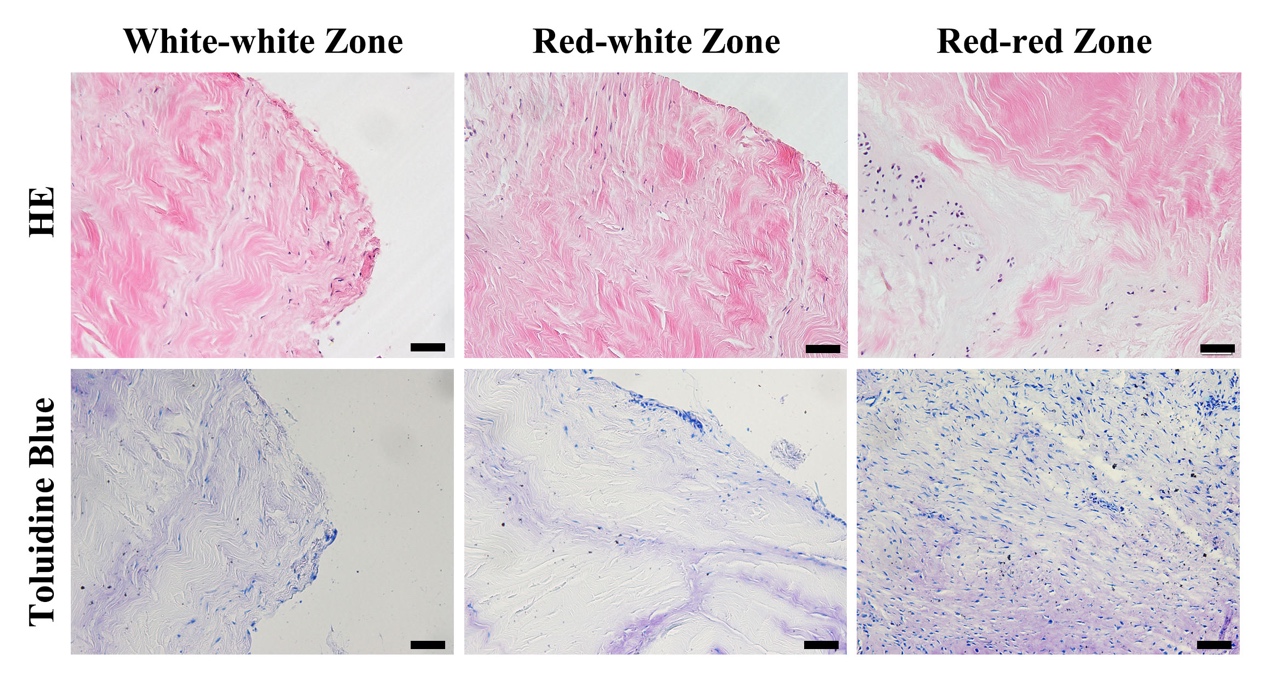


Supplementary Figure S5. HE staining and toluidine blue staining of regenerated meniscus at 6 weeks postoperative of transplantation group in white-white zone, red-white zone and red-red zone. Scale bar = 50 μm.


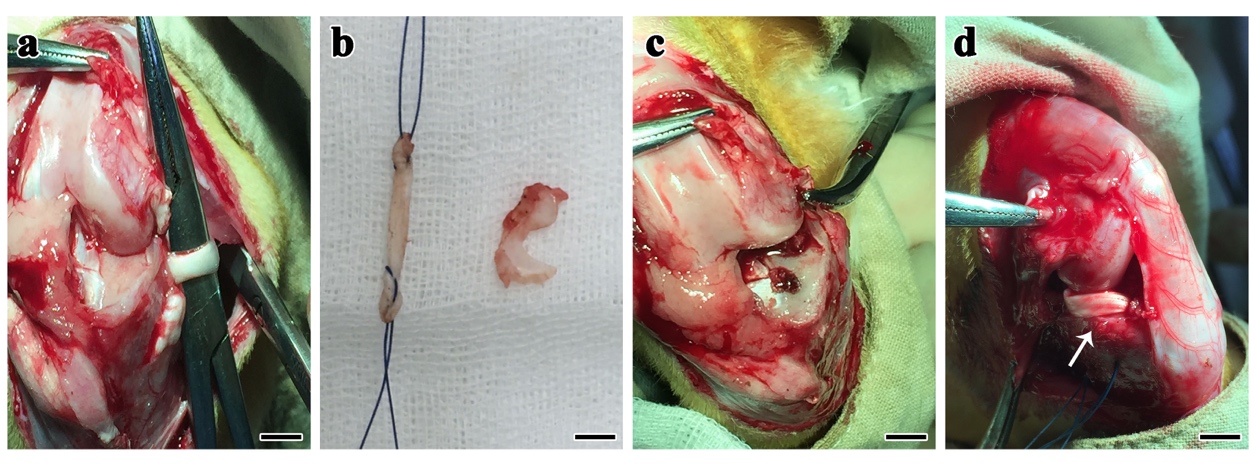
 Supplementary Figure S6. Surgical procedures of meniscus reconstruction in rabbits. Scale bar = 1cm. (a), Harvest of semitendinosus tendon. (b), Weaved tendon graft. (c), Bone tunnels drilled at insertion sites of the original medial meniscus on the tibia. (d), Tendon graft passed through the bony tunnel (white arrow, tendon graft).

Supplementary Table S1. The primer sequences used for real-time qPCR.

| Genes | 5’-3’ | Primer sequences |
| --- | --- | --- |
| *GAPDH* | Forward | GATTGTCAGCAACGCATCCTG |
|  | Reverse | TCCACAATGCCGAAGTGGTC |
| *SOX9* | Forward | CAAGAAAGACCACCCGGACT |
|  | Reverse | GCCTTGAAGATGGCGTTGGG |
| *COL2A1* | Forward | GGACGTTCAGGCGAAACTG |
|  | Reverse | CAAAGGCGCACATGTCGAT |
| *ACAN* | Forward | CCTACCAGGACAAGGTCTCG |
|  | Reverse | ACCTCACAGCGGTAGATCCC |
| *COL1A2* | Forward | AACGGTGCTCCTGGTGAAG |
|  | Reverse | GCGACCTGGAGGACCAT |
